# Validation of NINDS-VCI Neuropsychology Protocols for Vascular Cognitive Impairment in Taiwan

**DOI:** 10.1371/journal.pone.0156404

**Published:** 2016-06-01

**Authors:** Hsiu-Fen Lin, Chang-Ming Chern, Hui-Mei Chen, Yi-Chun Yeh, Shu-Chih Yao, Mei-Feng Huang, Shuu-Jiun Wang, Cheng-Sheng Chen, Jong-Ling Fuh

**Affiliations:** 1 Department of Neurology, Kaohsiung Medical University Hospital, Kaohsiung, Taiwan; 2 Department of Neurology, College of Medicine, Kaohsiung Medical University, Kaohsiung, Taiwan; 3 Department of Neurology, Neurological Institute, Taipei Veterans General Hospital, Taipei, Taiwan; 4 Faculty of Medicine, National Yang-Ming University School of Medicine, Taipei, Taiwan; 5 Graduate Institute of Medicine, College of Medicine, Kaohsiung Medical University, Kaohsiung, Taiwan; 6 Department of Occupational Therapy, College of Health Science, Kaohsiung Medical University, Kaohsiung, Taiwan; 7 Department of Psychiatry, Kaohsiung Medical University Hospital, Kaohsiung, Taiwan; 8 Department of Psychiatry, College of Medicine, Kaohsiung Medical University, Kaohsiung, Taiwan; University of Glasgow, UNITED KINGDOM

## Abstract

**Objective:**

To validate the three time-difference neuropsychological protocols developed by the National Institute of Health/National Institute of Neurological Disorders and Stroke (NINDS) and the Canadian Stroke Network for assessment of vascular cognitive impairment (VCI) in Mandarin-speaking subjects and to investigate the clinical application of the shortest form.

**Methods:**

Patients aged 50 years or older who had a stroke were invited to participate in the study. Clinical diagnosis of VCI was made. The NINDS-VCI Neuropsychology Protocols, 60-, 30-, and two 5-minute protocols, were administered. The criteria validities of the cognitive protocols against the diagnoses of stroke and VCI were determined via Receiver Operating Characteristic (ROC) analysis. The optimal cut-off point for the 5-minute protocols total score was estimated for clinical use in screening.

**Results:**

Eighty-three patients and 53 controls were recruited during the study period. Patients with stroke performed more poorly than the control group in the three neuropsychological protocols. Forty-two patients with stroke were diagnosed with VCI. VCI was used as the standard to estimate the criteria validities. The area under the ROC curve was 0.78, 0.80, 0.75, and 0.73 for the 60-, 30-, 5-mintue protocol-A and 5-minute protocol-B, respectively.

**Conclusion:**

These modified neuropsychological protocols can be used as valid instruments when performing comprehensive cognitive assessment or for screening of VCI in Taiwan.

## Background

Cognitive impairment affects up to half of stroke survivors [1]. Postmortem studies have indicated that up to two-thirds of patients with dementia exhibit brain vascular pathology [2]. These studies suggested that vascular lesions play a causative role in the development of cognitive impairment. Cerebrovascular disease may predispose, precipitate or perpetuate some cognitive impairment, which has been termed “vascular cognitive impairment” (VCI) [3]. As there are heterogeneous cerebrovascular vascular pathologies, the clinical presentation and course of VCI are varied. VCI can affect functional disability [4] and is a strong predictor of death [5]. It poses a huge economic and social burden in developed countries [6]. As vascular risk factors or diseases are modifiable or treatable[4], early recognition and identification of VCI is of great importance in the management of these patients.

Assessment of VCI requires valid neuropsychological protocols that are sensitive to specific cognitive deficits in the context of vascular etiologies. Current tools in use for cognitive function assessment were developed in the main based on the cognitive patterns specific to Alzheimer’s disease (AD). Evidence from previous studies has clearly indicated that there are cognitive differences between AD and VCI [4, 7]. Generally, VCI patients have greater executive dysfunction and slowing of mental speed but better memory than AD patients. There is a need to develop a cognitive instrument specific to patients with CVD, which has adequate sensitivity for the detection of cognitive impairment associated with vascular etiologies [4]. The National Institute of Health/National Institute of Neurological Disorders and Stroke (NINDS) and the Canadian Stroke Network sponsored a VCI Harmonization Workshop and developed standard protocols taking into account areas of neuropsychological research [3]. Three neuropsychology protocols, namely, 60- minute, 30-minute and 5-minute protocols for use in different settings for the assessment of VCI, have been proposed by the Neuropsychology Working Group. The 60-minute protocol was prepared for use in circumstances that require detailed cognitive assessment. It is constructed with four domains: executive function, language, visuospatial function and memory. Each domain is composed of several individual subtests. The 30-minute protocol is used for screening at clinics specializing in cognitive impairment. Tests were selected from within the 60-minute protocol. The 5-minute protocol was designed for potential use by primary clinics or community services, where quick screening is needed. Administration of the 5-minute protocol by telephone interview has the potential for use in the community. As there exist language differences, the neuropsychological protocols require modification in accordance with each society, and the validity of all three protocols must be established before they can be considered useful for the diagnosis of VCI in patients who speak different languages.

In this study, the set of three protocols was translated and modified into Mandarin Chinese. The purpose of this study was to validate these protocols for use in cognitive assessment among patients with stroke. Furthermore, the criteria validities of these three protocols were examined by determining the ability of these protocols to differentiate CVD patients with VCI from those without.

## Subjects and Methods

This study was conducted in outpatient and inpatient units of two teaching hospitals in Taiwan. Patients with stroke were invited to participate in the study if they were aged 50 years or older and had suffered a stroke within the past 3–18 months, defined according to the NINDS Stroke Data Bank criteria [8], with neuroimaging evidence of symptomatic cerebral infarction and at least one focal symptom and/or sign of stroke in the acute phase. Sufficient language skills and motor function for completion of neuropsychological tests were required. An informant, who spent at least 10 hours per week with the patients, knowledgeable about the subject’s recent and past medical history was required. The following causes of cognitive impairment were criteria for exclusion: (1) comorbidity with other brain diseases, such as neurodevelopmental and neurodegenerative diseases, hypoxic/anoxic states, hypotension or hypertension encephalopathy, inflammation, infection, epilepsy, and brain tumor; (2) an acquired metabolic, endocrine, traumatic, nutritional, or toxic disorder that could be judged to be affecting the brain; (3) major psychiatric disorder, such as substance use disorder, schizophrenia, and bipolar disorder; and (4) having diagnosis of dementia before the event of stroke.

Diagnosis of VCI was made according to observation of cognitive changes after stroke made by one knowledgeable informant of each patient. The research neurologist or psychiatrist interviewed each study participant and informant simultaneously using structural study interview schedule for diagnosis of cognitive disorders. The cognitive changes might involve remembering recent events, making decisions, handling personal matters, or language expression and comprehension. If the situation informants observed worsening of the patient’s condition, research physicians confirmed whether the changes came after the stroke according to the Diagnostic and Statistical Manual-5 (DSM-5) for neurocognitive disorder.

A control sample consisting of adults of similar age to the stroke sample was also studied. The inclusion criteria for these subjects were as follows: (1) no history of stroke or transient ischemic attack; (2) aged 50 or older; (3) possession of sufficient language skills to complete neuropsychological tests; (4) provision of written consent; and (5) achievement of a score of 24 or higher on the MMSE. The exclusion criteria were similar to those for the patient group.

The protocol was approved by the Institutional Review Boards of Taipei Veterans General Hospital and Kaohsiung Medical University Hospital. Written informed consent was obtained from all subjects prior to interview. If the participants have a compromised capacity to consent, their legally authorized family will provide written consents on the behalf of participants.

All subjects provided demographic information (sex, age, number of years of education and handedness), stroke history (duration of stroke illness) and medical and personal history. The National Institutes of Health Stroke Scale (NIHSS) was used to represent stroke severity [9]. Study participants received neuropsychological tests in the sequence of 60-minutue protocol, 5-minute protocols and MMSE. The data of 30-minute was derived from data of 60-minute one. Two clinical neuropsychologists, who were blind to the diagnosis of VCI, preformed the neuropsychological tests.

### The NINDS-VCI Neuropsychology Protocols

The 60-minute protocol was composed of four domains, within which there were several individual tests.

#### Executive Domain

Animal Naming [10]: Animal naming was used to assess category fluency. The score is the number of correct animal names produced in one minute.

WAIS-III Digit Symbol-Coding [11]: This is a brief timed coding test that is easy to administer. The score is the number of correct symbol-number pairs produced in two minutes.

Trail-making Test A part [12]: This is a short test involving working memory and set shifting. It is scored in terms of time to completion and number of errors.

#### Language Domain

Boston Naming Test (BNT) 2^nd^ edition, Short Form [13]: This is the most frequently used confrontation naming test. The score is the number of items correctly named.

#### Visuospatial Domain

Rey-Osterrieth Complex Figure Copy [14]: This test evaluates visuospatial abilities and executive function. A basic 36-item score based on location and accuracy is obtained [15], and an additional method allows for scoring of executive function [16].

#### Memory Domain

Hopkins Verbal Learning Test-Revised (HVLT-R)[17]: This is a 12-item supra-span list-learning task including three learning trials, a 20–25-minute delayed recall condition and a delayed recognition task. Three scores are generated, which represent the sum of items recalled across the three learning trials, the number of items recalled upon delay, and the recognition discrimination index.

Boston Naming Test Recognition [13]: A 20–25-minute delayed forced-choice recognition test for the BNT is administered, with an additional 15 items from the test as foils. The score is the number of pictures correctly recognized.

Digit Symbol-Coding Incidental Learning [18]: This includes free-recall drawing of the symbols used, followed by recall of matched number-symbol pairs. The two recorded scores are the number of symbols correctly recalled and correctly matched.

Complex Figure Memory [14]: This test includes free-recall drawing of a complex figure 20–25 minutes after viewing the initial copy.

The following sub-set of tests included in the 60-minute protocol is included in the 30-minute protocol: Animal naming, Digit Symbol-Coding, and HVLT-R. These test results were analyzed as a separate unit.

We created two versions of the 5-minute protocols. The first one, 5-minute protocol-A, consists of three subtests of the Montreal Cognitive Assessment (MoCA), and the Taiwanese version of the MoCA was validated previously by our group [19, 20]. It consists of a 20-point scale, with the total score computed as the sum of a 5-word delayed recall (5-point), a 6-item orientation (6-point), and the animal fluency test (0.5 points for each correct exemplar generated within one minute; maximum score 9 points) of the MoCA. The second test, the 5-minute protocol-B, replaced the orientation test with the WAIS-III Digit Symbol-Coding test. The rationale for the replacement is that Digit Symbol-Coding test is sensitive to brain lesions involving vascular pathology [21, 22]. It should be suitable to be include a brief screening tool. Raw scores of the WAIS-III Digit Symbol-Coding test were transformed to scaled scores [21] based on age. Then, 0.5 points were given for each scaled score, with a maximum score of 6 points, for the WAIS-III Digit Symbol-Coding test. The 5-minute protocol-B also yielded a maximum total score of 20. Both of these brief tasks were chosen because they assess both memory and executive function and can be used in a variety of languages and cultures.

The 30-point Mini-Mental State Examination (MMSE)[23] is a popular general cognitive screening examination, and was used for assessment of cognitive function in this study. This test assesses orientation, registration, attention, calculation, language, and recall, and has been widely used to assess elderly people. The total score ranges from 0 to 30, with a lower score indicating poorer cognitive function.

The Geriatric Depression Scale (GDS), first created by Yesavage, et al. [24], has been translated in Taiwanese [25]. We used the Short Form GDS which consists of 15 questions. The Short Form is more easily used by physically ill who may have short attention spans or feel easily fatigued.

The scores were then converted to standardized z scores, based on the scores of the control sample. For the 60-minute protocol, the z scores for the individual tests were grouped to form four domain z scores (executive, language, visuospatial function and memory), and an overall 60-minute summary z score was obtained as the average of these four z scores. One summary z score of the 30-minute protocol was computed with a single memory z score as the average of three HVLT z scores, the Animal Naming z score, and the Digit Symbol z score. Missing data will be excluded when calculating the average of each domain score. The raw total score was computed for the 5-minute protocols.

### Statistical Analysis

Student t tests or chi-square analyses were used for group comparisons. When the expected values in any of the cells of a 2 by 2 contingency table are below 5, we will use Fisher exact tests for comparisons. Correlations of two continuous variables were analyzed via Pearson’s correlation analysis. Statistical analyses were performed using the SPSS 14.0 statistical program, and the statistical significance level was set as p <0.05. We examined the external validity of the protocol according to how well its summary score can discriminate patients from controls using receiver operating curve (ROC) analysis. The criteria validities of the cognitive protocols against VCI were determined via ROC analysis [26]. Areas under the receiver operating characteristic curve (AUC) were compared using the ROCCONTRAST option of the logistic regression procedure of the SAS software program. The optimal cut-off point for the 5-minute protocol total score was determined using Youden-Index for which (sensitifity + specificity) is maximal [27, 28]. Accordingly, criteria validity indices were estimated with respect to sensitivity, specificity, positive predictive value (PPV), and negative predictive value (NPV).

## Results

### Demographic and clinical data

Eighty-three patients and 53 controls were recruited during the study period. The mean age of the stroke patients was 66.6 ± 9.7 years, and the education level was 9.2 ± 4.8 years. Males accounted for 61.9% (n = 52) in this cohort. The mean NIHSS of the stroke patients at admission was 2.2 (SD = 2.4). The mean number of days from onset of the index stroke to cognitive assessment was 9.0 ± 5.4 months. The numbers of patients with various physical conditions were as follows: smoking 23 (27.7%), alcohol drinking 5 (6.0%), hypertension 71 (85.5%), diabetes mellitus 39 (47.0%), hyperlipidemia 42 (50.6%), myocardial infarction 7 (8.4%), atrial fibrillation 11 (13.0%), heart failure 4 (4.8%), and heart valve disease 1 (1.2%). The demographic and clinical data are shown in table 1. Differences between the patients and controls were noted in terms of education level and for the physical conditions of smoking, hypertension, diabetes mellitus, and atrial fibrillation. A brief description of the neuroimaging features of the stroke patients is provided in table 2. Three of the stroke patients (3.6%) were cases of intracerebral hemorrhage, and the remainder, ischemic stroke patients.

**Table 1:** Group comparisons of demographic and clinical variables between the stroke patients and control subjects.

Variable	Controls (n = 53)	Stroke patients (n = 83)	p value
Age	66.1 (8.1)	66.6 (9.7)	0.761
Male	24 (45.3%)	52 (61.9%)	0.065
Education (years)	11.2 (3.2)	9.2 (4.8)	0.004
Physical conditions			
Smoking	3 (8.3%)	23 (27.7%)	0.019
Alcohol drinking	4 (11.1%)	5 (6.0%)	0.335
Hypertension	19 (52.8%)	71 (85.5%)	<0.001
Diabetes mellitus	6 (16.7%)	39 (47.0%)	0.002
Hyperlipidemia	14 (38.9%)	42 (50.6%)	0.24
Myocardial infarction	3 (8.3%)	7 (8.4%)	0.986
Atrial fibrillation	0 (0%)	11 (13%)	0.033
Heart failure	0 (0%)	4 (4.8%)	0.313
Heart valve disease	0 (0%)	1 (1.2%)	1.0
GDS	3.2 ± 3.1	3.9 ± 3.7	0.26

GDS: Geriatric Depression Scale

**Table 2:** Localization of lesions of stroke patients.

Location	n (%)
Frontal	
Left only	11 (13.3%)
Right only	8 (9.6%)
Bilateral	1 (1.2%)
Parietal-occipital	
Left only	4 (4.8%)
Right only	5 (6.0%)
Basal ganglia and subcortical white matter	
Left only	20 (24.1%)
Right only	13 (15.7%)
Thalamus	
Left only	2 (2.4%)
Right only	2 (2.4%)
Bilateral	1 (1.2%)
Brainstem	14 (16.9%)
Cerebellum	2 (2.4%)

All measures regarding cognitive function, both individual tests and domain scores, were lower in the patients with stroke than in the controls. The protocol summary scores of the 60-minute, 30-minute and 5-minute protocols were also all lower in the patient group (table 3).

**Table 3:** Means and standard deviations of neuropsychological measures in stroke patients and control subjects.

Protocol domain/individual test score mean (SD)	Controls (n = 53)	Stroke patients (n = 83)	p value
Executive			
Trail-making (sec)	54.7 ± 26.8	94.4 ± 66.7	<0.001
SDMT	43.0 ± 15.3	21.3 ± 13.1	<0.001
Verbal fluency	16.4 ± 4.8	11.4 ± 4.6	<0.001
Domain z score	0 ± 0.87	-1.33 ± 1.22	0.036
Language			
mBNT	13.8 ± 1.3	12.4 ± 2.2	<0.001
Domain z score	0 ± 0.99	-1.14 ± 1.70	<0.001
Visuospatial			
RCFT copy	30.2 ± 4.4	24.4 ± 10.3	<0.001
Domain z score	0 ± 1.00	-1.32 ± 2.34	<0.001
Memory			
HTLV-R learning	22.3 ± 5.1	16.0 ± 6.1	<0.001
HTLV-R delayed recall	6.5 ± 2.9	3.3 ± 3.1	<0.001
HTLV-R delayed recognition	11.5 ± 3.4	9.2 ± 3.1	<0.001
RCFT delayed recall	16.0 ± 8.7	12.3 ± 10.3	0.003
Domain z score	0 ± 0.66	-0.86 ± 0.89	<0.001
Supplementary			
MMSE	27.4 ± 2.6	25.0 ± 4.2	<0.001
Protocol summary score			
60-minute	0 ± 0.68	-1.16 ± 1.32	<0.001
30-minute	0 ± 0.82	-1.24 ± 0.81	<0.001
5-minute-A	16.7 ± 2.7	13.1 ± 3.8	<0.001
5-minute-B	13.2 ± 2.3	9.4 ± 3.2	<0.001

BNT: Boston Naming Test; RCFT: Rey-Osterrieth Complex Figure; HTLV-R: Hopkins Verbal Learning Test-Revised; MMSE: Mini-mental State Examination.

### Convergent validity

Correlations between each protocol and the MMSE were examined, and the results revealed that the Pearson correlation coefficients were 0.75 (p <0.001) for the 60-minute protocol, 0.75 (p <0.001) for the 30-minute protocol, and 0.70 (p <0.001) for both of the 5-minute protocols.

### Criteria validities to differentiate stroke patients and controls

ROC analyses were used to represent the external validities of these protocols to differentiate patients with stroke from controls. The area under the ROC curve was 0.78 (95% CI = 0.70–0.87; p <0.001) for the 60-minute protocol, 0.85 (95% CI = 0.77–0.92; p <0.001) for the 30-minute protocol, 0.78 (95% CI = 0.70–0.86; p <0.001) for the 5-minute protocol-A, and 0.83 (95% CI = 0.75–0.90; p <0.001) for the 5-minute protocol-B. Comparisons of the AUCs of the three protocols revealed that the AUC of the 30-minute protocol was greater than that of the 60-minute protocol (p = 0.029) and that of the 5-minute protocol-A (p = 0.007). Other comparisons of AUCs did not show statistical significance (p = 0.83 for 60-minute vs 5-minute-A; p = 0.20 for 60-minute vs 5-minute-B; p = 0.46 for 30-minute vs 5-minute-B; and p = 0.10 for 5-minute-A vs 5-minute-B).

### Criteria validities to differentiate VCI and non-VCI among patients with stroke

Forty-two patients with stroke were diagnosed with VCI, including 13 with vascular dementia, based on our research criteria. Their demographic, clinical and cognitive characteristics are shown in table 4. Compared with the patients without cognitive impairment, there were no significant statistical differences in the demographic and clinical variables except educational level, whereas all cognitive assessment results, including individual tests and domain z scores, were poorer among the patients with VCI.

**Table 4:** Means and standard deviations of neuropsychological measures in stroke patients with and without VCI.

	Cognitively-intact stroke patients (n = 41)	VCI patients (n = 42)	p value
Age	64.5 (8.5)	68.5 (10.5)	0.06
Male	27 (65.9%)	24 (57.1%)	0.415
Education (years)	10.3 (5.0)	8.1 (4.4)	0.03
Smoking	13 (31.7%)	10 (23.8%)	0.422
Alcohol drinking	3 (7.3%)	2 (4.8%)	0.676
Hypertension	34 (82.9%)	37 (88.1%)	0.503
Diabetes mellitus	20 (48.8%)	19 (45.2%)	0.746
Hyperlipidemia	23 (56.1%)	19 (45.2%)	0.322
Myocardial infarction	4 (9.8%)	3 (7.1%)	0.713
Atrial fibrillation	5 (12.2%)	6 (14.3%)	0.779
Heart failure	2 (4.9%)	2 (4.8%)	1.0
Heart valve disease	0 (0%)	1 (1.2%)	1.0
Executive			
Trail making (sec)	69.1 ± 37.4	119.8 ± 79.3	0.001
Digit symbol-coding	26.4 ± 11.9	16.4 ± 12.3	<0.001
Verbal fluency	13.0 ± 3.6	9.8 ± 5.0	0.001
Domain z score	-0.79 ± 0.78	-1.85 ± 1.34	<0.001
Language			
BNT	13.4 ± 1.4	11.3 ± 2.3	<0.001
Domain z score	-0.32 ± 1.11	-1.95 ± 1.81	<0.001
Visuospatial			
RCFT copy	28.8 ± 7.1	20.1 ± 11.3	<0.001
Domain z score	-0.33 ± 1.61	-2.28 ± 2.55	<0.001
Memory			
HTLV learning	18.9 ± 4.7	13.1 ± 5.9	<0.001
HTLV delayed recall	4.7 ± 3.2	2.2 ± 2.7	0.005
HTLV delayed recognition	10.6 ± 1.9	7.8 ± 3.4	<0.001
RCFT delayed recall	14.6 ± 10.4	7.5 ± 8.9	0.001
Domain z score	-0.37 ± 0.69	-1.34 ± 0.81	<0.001
Supplementary			
MMSE	27.1 ± 2.7	22.8 ± 4.3	0.001
Protocol summary score			
60-minute	-0.45 ± 0.85	-1.85 ± 1.33	0.001
30-minute	-0.82 ± 0.62	-1.64 ± 0.77	0.001
5-minute-A	14.8 ± 2.9	11.4 ± 3.8	<0.001
5-minute-B	10.8 ± 2.7	8.3 ± 3.1	<0.001

BNT: Boston Naming Test; RCFT: Rey-Osterrieth Complex Figure; HTLV-R: Hopkins Verbal Learning Test-Revised; MMSE: Mini-mental State Examination.

For patients with stroke alone, the criteria validities of the 60-, 30-, and 5-mintue protocols to differentiate patients with VCI from stroke patients without VCI were assessed. The area under the ROC curve was 0.78 (95% CI = 0.68–0.89; p <0.001) for the 60-minute protocol, 0.80 (95% CI = 0.69–0.90; p <0.001) for the 30-minute protocol, 0.75 (95% CI = 0.63–0.86; p <0.001) for the 5-minute protocol-A and 0.73 (95% CI = 0.61–0.85; p = 0.002) for the 5-minute protocol-B. The p value of each pairwise comparison of the AUCs of all protocols did not reach statistical significance (p = 0.66 for 60-minute vs. 30-minute;, p = 0.50 for 60-minute vs. 5-minute-A; p = 0.25 for 60-minute vs. 5-minute-B; p = 0.16 for 30-minute vs. 5-minute-A;, p = 0.13 for 30-minute vs. 5-minute-B; p = 0.61 for 5-minute = A vs. 5-minute-B).

### Criteria validity indices for the all protocols

The criteria validities in terms of sensitivity, specificity, PPV, and NPV for the 60-minute, 30-minute and both 5-minute protocols were examined. To differentiate patients with stroke and controls, the 60-minute protocol had a sensitivity of 78.3%, a specificity of 67.9%, a PPV of 79.3%, and a NPV of 66.7% at the optimal cut-off point of -0.16 z score. The 30-minute protocol had a sensitivity of 89.2%, a specificity of 71.7%, a PPV of 83.1%, and a NPV of 80.9% at the optimal cut-off point of -0.34 z score. As the total scores of the 5-minute protocols could be summed directly, we suggest that the optimal cut-off point for the 5-minute protocol-A is 15/16, which had a sensitivity of 73.1%, a specificity of 64.2%, a PPV of 72.1%, and a NPV of 65.4%. The 5-minute protocol-B had a sensitivity of 82.1%, a specificity of 67.9%, a PPV of 76.4%, and a NPV of 75.0% at the cut-off point of 12/13.

When differentiating stroke patients with VCI from stroke patients without VCI, the 60-minute protocol had a sensitivity of 73.8%, a specificity of 75.6%, a PPV of 75.6%, and a NPV of 73.8% at the optimal cut-off point of -0.85 z score. The 30-minute protocol had a sensitivity of 71.4%, a specificity of 68.3%, a PPV of 69.8%, and a NPV of 70.0% at the optimal cut-off point of -1.15 z score. The optimal cut-off point for the 5-minute protocol-A was 14/15, which had a sensitivity of 80.6%, a specificity of 54.8%, a PPV of 67.4%, and a NPV of 70.8%. The 5-minute protocol-B had a sensitivity of 75.0%, a specificity of 61.3%, a PPV of 69.2%, and a NPV of 67.9% at the cut-off point of 10/11.

## Discussion

The results of this study showed that all three Mandarin NINDS-VCI Neuropsychology Protocols had a good validity for the differentiation of patients with VCI from those without. All three protocols presented a good convergent validity with the MMSE. Using two 5-minute protocols for VCI screening purposes, there were fair to good criteria validity indices, including sensitivity, specificity, PPV, and NPV.

There have been various Chinese versions of NINDS-VCI neuropsychological batteries developed in Asian, such as in Hong Kong [29] and China [30]. Including ours, the main change in these versions from original version were the replacement of the test to assess letter fluency with one to assess category fluency. Given the considerable involvement of language in the verbal fluency test, it is likely infeasible that letter verbal fluency can be directly applied in the Chinese context. As both phonemic and semantic verbal fluency deficits in vascular dementia may relate to a similar effortful retrieval requirement, and patients with small vessel disease exhibit similar impairments in both letter and category fluency tasks [31], the satisfactory psychometric properties of this Mandarin version suggest the feasibility of this modification. Another change was that the Trail-Making Test Part B was not adapted in this study, as a substantial proportion of the elderly in Taiwan is illiterate. The differences within the three Chinese versions were mainly in the 5-minute protocols. In our study, the 5-minute protocol-A did not include immediate (5 points) and recognition memory (5 points), unlike the other two Chinese versions. The 5-minute protocol-B replaced the orientation part (6 points) with the WAIS-III symbol digit test (6 points). Our two 5-minute protocols placed more weight on tests involving executive function and processing speed. Including our version, all Chinese versions of 5-minute protocols exhibited fair to good accuracy in differentiating stroke patients from controls. There were large effect sizes of difference across all cognitive measurements assessed, not only in differentiating stroke patients from healthy controls, but in differentiating stroke patients with VCI from those without. The effect sizes can be represented with high AUCs of 60-, 30-, and 5-minute protocols (0.78, 0.85, 0.78 in differentiating stroke patients; and 0.85, 0.88, 0.82 in differentiating VCI patients). These results of effect sizes were similar with two previous studies [29, 30], which demonstrated adequate discriminatory power. The findings supported the validity of these 3 protocols for cognitive assessment of patients with stroke, and moreover, for patients with VCI. The 60-minute protocol has the advantage of yielding an assessment of breakdown of cognitive performance across various cognitive domains. The pattern of cognitive impairment is dependent on the locations involved in the stroke, especially within the cortical area. A comprehensive assessment may cover all patterns of VCI. The use of the 60-minute protocol has considerable benefits for comprehensive measurement of cognitive status or cognitive change in clinical assessment. For the 30-minute protocol, the effect size of difference between cases and controls and the ROC AUC value appear larger than those for the 60-minute protocol, which may be explained by the fact that the content of the 30-minute protocol focuses more on executive dysfunction and slowing of mental speed associated with vascular lesions. As both protocols have distinct advantages, we suggested that which protocol is selected in use dependent on how much time available at clinical assessment.

The results of this study supported the usefulness of the 5-minute protocol in the primary clinical setting. In the primary clinical setting, it is important to identify individuals with cognitive complaints related to objective impairment. The 5-minute protocol is more simple to perform than the other two protocols, and the total score without standardization is easily obtained in the primary clinical setting. Our findings of a fair to good criteria validity indicated that the 5-minute protocol can differentiate subjects with VCI from those without, even for the target population with stroke. Among the criteria validity indices, we obtained a good sensitivity and NPV, whereas the specificity and PPV only showed fair values. A relatively large portion of stroke patients without VCI scored low of the 5-minute protocol might explain the fair specificity and PPV. That is, the false positives were high. In fact, patients with stroke may present with slowing of psychomotor activities, even without significant cognitive impairment. Sometimes, they may perform below their ability when completing a new neuropsychological test. Being allowed more time to complete tests may reduce the false positive rate. Beyond the caveats, according to the nature of free of pencil and paper tests, further studies can be conducted to examine their feasibility by phone administration.

Some study caveats have to be addressed with reference to standards for studies of diagnostic test accuracy in dementia [32]. Diagnosis of VCI in this study was made based mainly on cognitive decline and not impairment of activities of daily living; that is, patients with VCI may include those with a diagnosis of vascular dementia. Another diagnostic issue was that neuropsychological tests, in general, are more sensitive to detect mild cognitive deficits as they are present in VCI than informant information which rather capture more obvious and severe deficits. Although the rate of VCI of these study sample was compatible to those in the literature, it is still need to be addressed for the diagnostic limitation in this study. As the study participants were not community-dwelling residents, and the validity is dependent on the prevalence of VCI in the specific setting, misclassification, including false positives, of subjects using this tool is possible. As calculating predictive values is problematic based on the prevalence from case-control data, the PPV and NPV of this case-control study should be interpreted cautiously interpreted. Generalization to other societies should be performed cautiously. Second, there is still variability even within Chinese-speaking societies, and therefore cross-culture validation in other settings and populations is necessary. Additionally, the distribution of cases in this study was mainly restricted to infarction patients and those with sufficient language skills and motor function to complete the neuropsychological tests; more information about other stroke patients is needed. Third, all study participants underwent neuropsychological tests in the following sequence: 60-minute protocol, 5-minute protocol-A, and MMSE. Data for the 30-minute protocol and the 5-minute protocol-B were derived, rather than assessed directly. It is possible that the results would exhibit minor differences if the sequence of administration was not exactly the same for each patient. Fourth, those with low motivation and a low willingness to participate in the study were excluded. As a significant proportion of patients who have suffered a stroke have depressive symptoms, we decided not to exclude patients with higher GDS scores. However, a possible confounding effect of depression cannot be ruled out. Finally, most diagnostic tests require large samples to increase the power estimates.

## Conclusion

We found that all three Mandarin NINDS-VCI Neuropsychology Protocols had a good validity to differentiate patients with VCI from those without. The three protocols are suggested to be used in various settings and for different assessment purposes, such as comprehensive cognitive assessment or for screening of VCI.
